# What are the views of Quebec and Ontario citizens on the tiebreaker criteria for prioritizing access to adult critical care in the extreme context of a COVID-19 pandemic?

**DOI:** 10.1186/s12910-024-01030-2

**Published:** 2024-03-19

**Authors:** Claudia Calderon Ramirez, Yanick Farmer, Andrea Frolic, Gina Bravo, Nathalie Orr Gaucher, Antoine Payot, Lucie Opatrny, Diane Poirier, Joseph Dahine, Audrey L’Espérance, James Downar, Peter Tanuseputro, Louis-Martin Rousseau, Vincent Dumez, Annie Descôteaux, Clara Dallaire, Karell Laporte, Marie-Eve Bouthillier

**Affiliations:** 1 Biomedical Sciences Program, Clinical Ethics, Faculty of Medicine, Université de Montréal, Pavillon Roger-Gaudry 2900 Bd Édouard-Montpetit, Montréal, Québec H3T 1J4 Canada; 2 Department of Social and Public Communication, Faculty of Communication, succursale Centre-Ville, Université du Québec à Montréal, Montréal, Québec C.P 8888, H3C 3P8 Canada; 3 Program for Ethics and Care Ecologies (PEaCE), Hamilton Health Sciences – King West, P.O. Box 2000, Hamilton, ON L8N 3Z5 Canada; 4 Department of Community Health Sciences, Faculty of Medicine and Health Sciences, Université de Sherbrooke, 2500 Bd de l’Université, Sherbrooke, Québec J1K 2R1 Canada; 5 Research Centre of the Sainte-Justine University Hospital, 3175 Chem. de la Côte-Sainte- Catherine, Québec, Montreal, H3T 1C5 Canada; 6 Office of Clinical Ethics, Faculty of Medicine, Université de Montréal, Pavillon Roger-Gaudry, 2900 Bd Édouard-Montpetit, Montréal, Québec H3T 1J4 Canada; 7 Executive Office Administration, Faculty of Medicine, McGill University Health Centre, Site Glen 1001 boul. Décarie, Montréal, Québec H4A 3J1 Canada; 8 CIUSSS du Centre-Sud-de-l‘Île-de-Montréal, 1560, rue Sherbrooke Est, Montréal, Québec H2L 4M1 Canada; 9 Department of Medicine and Medical Specialties, Faculty of Medicine, Université de Montréal, Pavillon Roger-Gaudry 2900 boulevard Édouard-Montpetit, Montréal, Québec H3T 1J4 Canada; 10 École nationale d’administration publique (ENAP), 4750 Av. Henri-Julien, Montréal, Québec H2T 2C8 Canada; 11 Division of Palliative Care, Department of Medicine, University of Ottawa, 43 Rue Bruyere St. 268J, Ottawa, ON K1N 5C8 Canada; 12 Ottawa Hospital Research Institute, 501 Smyth Rd, Box 511, Ottawa, ON K1H 8L6 Canada; 13 Faculty of Engineering, Montreal Polytechnic, Chem. de Polytechnique, Montréal, Québec 2500, H3T 1J4 Canada; 14 Centre d’Excellence sur le Partenariat avec les Patients et le Public (CEPPP) CRCHUM –, Pavillon S 850, rue St-Denis, porte S03.900, Montréal, Québec H2X 0A9 Canada; 15 Bureau du Patient Partenaire, Faculté de médecine, Université de Montréal, Pavillon Roger- Gaudry 2900 boulevard Édouard-Montpetit, bureau R-815, Montréal, Québec H3T 1J4 Canada; 16 Medical residency program, Faculty of Medicine, Université de Montréal, Pavillon Roger- Gaudry 2900 boulevard Édouard-Montpetit, Montréal, Québec H3T 1J4 Canada; 17 Department of Family and Emergency Medicine, Faculty of Medicine, Université de Montréal, 2900 Bd Édouard-Montpetit, Montréal, Québec H3T 1J4 Canada

**Keywords:** COVID-19 prioritization, Tiebreakers, Critical care, Democratic deliberation, Clinical ethics

## Abstract

**Background:**

The prioritization protocols for accessing adult critical care in the extreme pandemic context contain tiebreaker criteria to facilitate decision-making in the allocation of resources between patients with a similar survival prognosis. Besides being controversial, little is known about the public acceptability of these tiebreakers. In order to better understand the public opinion, Quebec and Ontario’s protocols were presented to the public in a democratic deliberation during the summer of 2022.

**Objectives:**

(1) To explore the perspectives of Quebec and Ontario citizens regarding tiebreakers, identifying the most acceptable ones and their underlying values. (2) To analyze these results considering other public consultations held during the pandemic on these criteria.

**Methods:**

This was an exploratory qualitative study. The design involved an online democratic deliberation that took place over two days, simultaneously in Quebec and Ontario. Public participants were selected from a community sample which excluded healthcare workers. Participants were first presented the essential components of prioritization protocols and their related issues (training session day 1). They subsequently deliberated on the acceptability of these criteria (deliberation session day 2). The deliberation was then subject to thematic analysis.

**Results:**

A total of 47 participants from the provinces of Quebec (*n* = 20) and Ontario (*n* = 27) took part in the online deliberation. A diverse audience participated excluding members of the healthcare workforce. Four themes were identified: (1) Priority to young patients - the life cycle - a preferred tiebreaker; (2) Randomization - a tiebreaker of last resort; (3) Multiplier effect of most exposed healthcare workers - a median acceptability tiebreaker, and (4) Social value – a less acceptable tiebreaker.

**Conclusion:**

Life cycle was the preferred tiebreaker as this criterion respects intergenerational equity, which was considered relevant when allocating scarce resources to adult patients in a context of extreme pandemic. Priority to young patients is in line with other consultations conducted around the world. Additional studies are needed to further investigate the public acceptability of tiebreaker criteria.

**Supplementary Information:**

The online version contains supplementary material available at 10.1186/s12910-024-01030-2.

## Background

Prioritization protocols for resource allocation in adult intensive care were designed to be applied in extreme cases of resource scarcity in the decision-making process of admission, and to avoid, as much as possible, arbitrariness in the distribution of finite resources. During the COVID-19 pandemic, the Quebec and Ontario health authorities developed prioritization protocols for patients requiring admission to critical care units in a context of extreme overcapacity [1, 2]. These prioritization models contemplated criteria based on ethical principles and values, emphasizing the maximization of benefits in a pandemic crisis. Like most prioritization protocols, the Quebec and Ontario protocols considered clinical criteria that predicted survival and outcomes to maximise the number of lives saved. However, when clinical criteria are insufficient to determine how to allocate intensive care resources, additional tiebreaker criteria can be considered for patients with similar prognosis [3, 4]. Among the tiebreakers most frequently encountered in the scientific literature during the COVID-19 pandemic were those related to the age of the patient both directly and indirectly (absolute age, life cycle and various terms of life years including “fair innings”), those associated with the instrumental value of patients and their role in society (the most exposed healthcare workers, pregnant women or caregivers of vulnerable people), those associated with random selection (lottery or coin toss), and those related to the ease of accessing healthcare services (“the first-come first-served” principle) [5–9]. Similar criteria had already been contemplated in other clinical protocols for dealing with previous pandemics [3, 10].

Most of these tiebreakers are controversial and continue to be debated, especially those relating to age, social value, and the prioritization of healthcare workers [11–13]. Results from online democratic deliberations (DD) held during the COVID-19 pandemic in England suggested that the participating public rejects the principles associated with life projects and “fair innings”. Participants justified this rejection by pointing out that some people flourish later in life, for example in retirement, and that the elderly are still useful [11]. Another DD in Thailand found that the public did not agree with considering the social value of individuals when deciding which patients should be allocated scarce resources in the context of a pandemic. Participants did not consider this criterion to be fair [12]. A third DD conducted to assess the acceptance of prioritizing healthcare workers, showed divergence in opinions between participants from the public and healthcare professionals. The former were willing to prioritize healthcare professionals, while healthcare professionals disagreed [13].

In view of the existing controversies related to the prioritization tiebreakers and the criticisms that have arisen towards the existing protocols, healthcare authorities and researchers considered it essential to conduct public DD to gauge their acceptability. DD favors two important procedural values to prevail: transparency and accountability [14, 15]. Citizen opinion must be considered, as they are the target audience for these new policies. DD provides opportunities for knowledge exchange between experts/policy makers and the public. In addition to highlighting the pluralism of ideas in the population, DD improves decision-making by generating new insights and solutions to existing problems. This informed opinion can then serve as the basis for the policy-making process.

The main purpose of this study was to know the participating public’s opinions regarding the acceptability of these tiebreakers, as well as to better understand the underlying values based on their pro and con arguments.

## Methods

### Research design overview

This study used a qualitative design. We chose to carry out an online democratic deliberation (DD) because this type of method would allow us to obtain feedback -a two-way learning-, between experts and non-experts under social distancing measures [16, 17]. DD provides information to non-expert participants on a specific topic to prepare them for their active contribution in the deliberation. It is suitable when the topic to be deliberated involves ethical issues that would benefit from prior discussions. For this reason, this methodology has been used more frequently for research in clinical ethics and bioethics [18]. This DD was not intended to reach a consensus among participants; the consensus would be optional. Our main research interests were to explore and to analyse the perspectives of the participating public regarding the acceptability of these protocols’ tiebreakers and how the public justifies their preferences and values.

#### Data collection and procedure

The DD took place over two days simultaneously for the Quebec and Ontario groups, in French and English respectively. Day 1, in May 2022, included a training session. Day 2, in June 2022 was the deliberative session. The preparation and logistics of this deliberation were jointly carried out by the Institut du Nouveau Monde (INM) and the research team. Details are provided in Additional file 1.

#### Training session day 1

The training sessions’ goal was to provide participants with information on the subject matter and to give them the opportunity to question the experts about the prioritization protocols. The training day took place on May 28, 2022, and lasted 8 h, including breaks and small group meetings. Both sets of experts shared with each other the content of their presentations to ensure they all provided the same content. In addition to the training session, an online information sheet was sent to all participants explaining, in general terms, the main ethical issues underlying the criteria considered in the protocols to deepen their understanding in this regard. Details are provided in Additional file 2.

#### Deliberative session day 2

The deliberation session lasted 4 h and was held on June 4, 2022. During this session we collected the qualitative data presented in this paper. Facilitators were present for each of the groups, who discussed and deepen with the participants their perspectives on tiebreakers, initially in a general way and then directly on their acceptability and unacceptability. For each tiebreaker, participants were asked to assign a score from 0 (complete unacceptability) to 5 (complete acceptability).

### Staff

#### Presenters

The training session presenters were 8 experts (4 in Quebec, 4 in Ontario) specialized in adult and pediatric critical care, ethics, anthropology, professionals working in partnership with patients, and university professors.

#### Facilitators

Two main facilitators, one for the French sessions and the other for the English sessions, oversaw the animation of plenary sessions. They were assisted by 14 other facilitators who took care of the small group sessions.

#### Observers

A total of 6 observers (3 observers in each group) consisted of students and clinical ethicists. A structured online observation sheet was provided to the observers so that they could take notes during the deliberation session. One of the observers was also available to assist participants in case they did not feel comfortable during the process.

### Recruitment process

#### Participant recruitment

This recruitment was carried out by Leger Opinion and the Institute du Nouveau Monde (INM). Both organizations have experience in promoting citizen participation and public deliberative processes. The selection of participants was initially carried out at random by Leger Opinion among the members of the public registered on their poll website. The INM then selectively sampled among these participants to ensure a diversity of participants considering the following criteria: origin, age, gender, educational level, income, language, functional limitations, and ethnicity. This selective sample included 60 participants from both provinces: 30 from Quebec and 30 from Ontario. Some participants could not access the online training session, due to internet failures caused by weather conditions. These participants did not continue in the deliberation process. For these reasons, the final sample was composed of 20 participants from Quebec and 27 participants from Ontario.

### Criteria for selecting participants

The selection criteria for participants were as follows:

- Individuals over 18 years of age.
- Individuals who were not currently working as professionals, students or technicians in healthcare or healthcare-related social services.
- Individuals from the provinces of Quebec and Ontario.
- Individuals fluent in French (Quebec) or English (Ontario).
- Canadian citizens.
- Individuals with basic physical and mental abilities to easily participate in the online sessions and with an access to the Internet.

The reason why participants were chosen based on these selection criteria was mainly to promote the participation of individuals with different profiles and with little or no experience with the healthcare system. Furthermore, only including non-healthcare professionals in the deliberation insured that participants’ opinions were not influenced by healthcare professionals with a higher knowledge about the topic to be deliberated.

### Recording and data transformation

The online deliberation was conducted through the Zoom platform. Participants’ opinions and statements were recorded and transcribed in English and French. Prior to the thematic analysis, the transcripts were de-identified to preserve the confidentiality of the participants by (MEB, CC). The transcripts were transferred to the *NVivo 2022 software* through which the data were organized, managed, and compared [19]. All data were securely stored in locked files and password-protected in the University’s OneDrive system.

### Analysis

A thematic analysis was conducted [20–22]. As a starting point, the coders (CC, KL) and supervisor (MEB) read the transcribed verbatim several times, letting emerge what the citizens said in terms of the acceptability of these tiebreakers, the arguments for and against them, and the values associated with them. They came up with an initial codebook that was then revisited and transformed following subsequent iterations of coding, grouping codes into themes, and re-evaluating the resulting coding schema. The researchers (CC, KL, MEB) discussed and challenged the coding list in an iterative process until consensus was reached. A final codebook was then used to re-evaluate the coding a final time. This process provided the opportunity to check that the coding, including the resulting themes, was an accurate representation of the deliberation session data. An inter-coder reliability was computed which indicated a percentage of agreement between the two coders (CC, KL) of ≥ 97% and an overall Kappa index of 0.8. Details are provided in Additional file 3.

## Results

A total of 47 participants attended the online DD held on June 4, 2022. There were 20 participants from the province of Quebec and 27 from the province of Ontario. Demographic data of participants are shown in [Table 1].

**Table 1:** Demographic variables of the deliberators (*N* = 47)

Variables	Quebec 20 (43%)	Ontario 27 (57%)
**Gender**		
Male Female Other	9 (45) 11 (55) 0 (0)	14 (52) 13 (48) 0 (0)
**Age**		
18–24 25–34 35–44 45–54 55–64 65–74 ≥ 75	3 (15) 1 (5) 3 (15) 6 (30) 2 (10) 4 (20) 1 (5)	2 (7.4) 3 (11) 5 (18.5) 5 (18.5) 5 (18.5) 4 (14.8) 3 (11)
**Region**		
	Bas-Saint-Laurent 1 (5) Saguenay–Lac-Saint-Jean 1 (5) Capitale-Nationale 4 (20) Estrie 1 (5) Montréal 3 (15) Outaouais 1 (5) Abitibi-Témiscamingue 1 (5) Côte-Nord 1 (5) Gaspésie–Îles-de-la-Madeleine 1 (5) Lanaudière 2 (5) Montérégie 3 (5) Centre-du-Québec 1 (5)	Centre 5 (18.5) East 4 (14.8) Greater Toronto 12 (44.4) Northeast 1 (3.7) Northwest 1 (3.7) Southwest 4 (14.8)
**First Nations**		
Yes	1 (5)	1 (3.7)
No	0	0
**Functional limitations**		
Yes	0	6 (22.2)
No	20 (100)	21 (77.7)
**Ethnocultural groups**		
Asian (e.g. Chinese, Korean, Japanese, Filipino) South Asian (e.g., Indian, Pakistani) Latin American Afro American Arab Visible minority Multiple visible minorities Not a visible minority I prefer not to answer	0 0 2 (10) 0 0 1 (5) 0 16 (80) 1 (5)	3 (11) 2 (7.4) 0 2 (7.4) 1 (3.7) 1 (3.7) 1 (3.7) 17 (62.9) 0
**Schooling**		
Primary (7 years or less) Secondary (DES in general or vocational training) College (DEC pre-university training, technical) University 1 (Bachelor’s degree) University 2 (Master’s degree) University 3 (Doctorate) I prefer not to answer	0 4 (20) 8 (40) 4 (20) 3 (15) 0 1 (5)	0 7 (25.9) 6 (22.2) 9 (33.3) 4 (14.8) 1 (3.7) 0
**Occupation**		
I am studying or in training I am working I am taking care of my children or a relative I am retired I am unemployed I am self-employed	0 13 (65) 1 (5) 3 (15) 2 (10) 1 (5)	1 (3.7) 11 (40.7) 0 9 (33.3) 3 (11) 3 (11)
**Income**		
Less than 30,000$ 30 000$ à 49 999$ 50 000$ à 69 999$ 70 000$ à 99 999$ 100 000$ and more	4 (20) 6 (30) 3 (15) 5 (25) 1 (5)	4 (14.8) 5 (18.5) 8 (29.6) 5 (18.5) 5 (18.5)

Source: INM data

### Online democratic deliberation

**Fig. 1:**
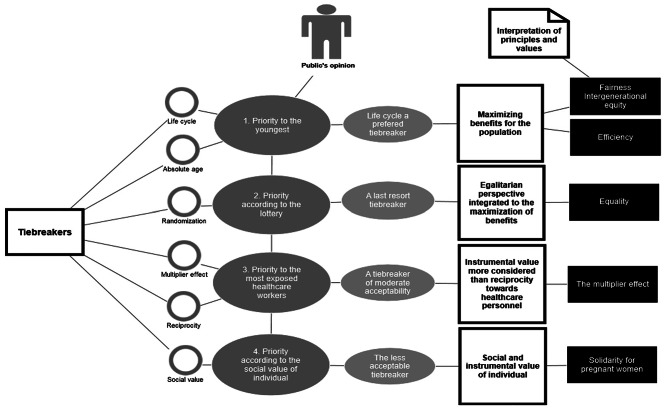
*NVivo 2022* Coding tree. Diagram showing the themes and values underlying the tiebreaker

The citizens of both Quebec and Ontario were questioned about their opinions regarding each tiebreaker criteria. They were asked to present their arguments for and against each of the tiebreakers. They were also asked to identify which tiebreakers were the most and least acceptable to them. Thus, our four emerging themes were centered on the acceptance or non-acceptance of the tiebreakers. Our main themes were: (1) Priority to the youngest - Life cycle a preferred tiebreaker; (2) Randomization - A tiebreaker of last resort; (3) The multiplier effect of the most exposed healthcare workers - A tiebreaker of moderate acceptability; and (4) Social value of individual – A less acceptable tiebreaker. A diagram -Coding tree *NVivo 2022*- was developed to show the main emerging themes and values underlying the tiebreakers according to our analysis and interpretation [Fig. 1]. This coding tree shows that, in general, the participating public considered it important to give priority to young patients through intergenerational equity, with the life cycle being a favorite tiebreaker. They also considered it important to manage scarce healthcare resources judiciously, especially in an extreme context, to streamline intensive care, and allow more patients to benefit. We observed, in general, a utilitarian perspective based on maximization for the common good. We also found other values that were considered important in prioritizing patients in case of a tie, such as the value of efficiency, equality, solidarity, and instrumental value.

**Fig. 2:**
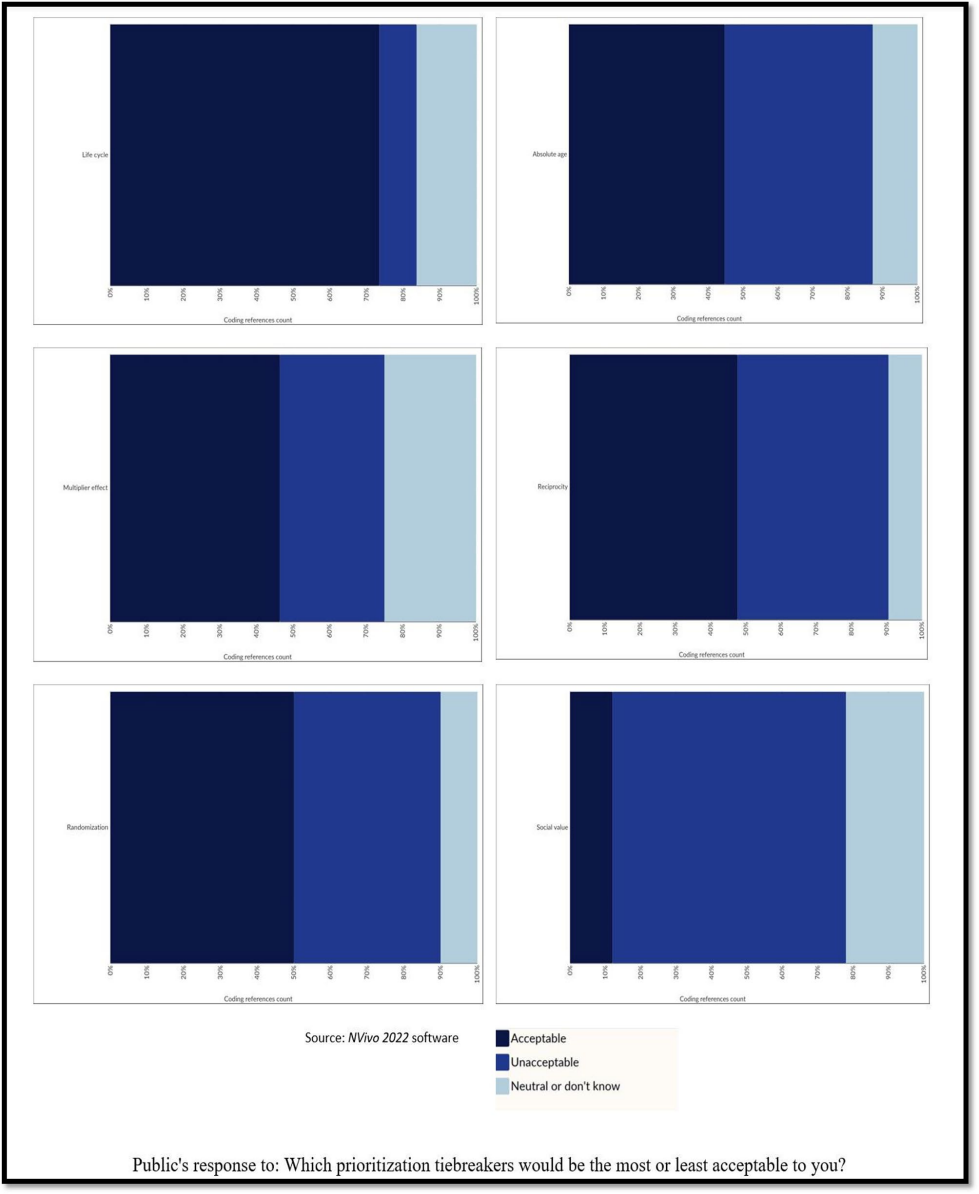
Public’s response to: Which prioritization tiebreakers would be the most or least acceptable to you?

The pattern found in the data coding regarding the acceptability and unacceptability of the tiebreakers by the public’s opinion is presented in Fig. 2. This representation allowed us to appreciate the level of support for each tiebreaker . The greatest convergence was observed in two tiebreakers: favoring the life cycle and disfavoring the social value of the individual. In contrast, we found a divergence of opinions among participants with the tiebreakers of absolute age and reciprocity. Regarding the tiebreaker of favoring healthcare personnel, the most exposed group, we noted that the value of reciprocity for frontline healthcare workers was poorly supported, as opposed to its multiplier effect. The multiplier effect or instrumental value was moderately accepted. In the case of randomization, its acceptance was present, but not predominant. Few participants did not know what to answer or were unsure of their responses when considering the acceptability of some the tiebreakers presented. In addition to these results, a coding comparison diagram showed that most of the public consulted preferred life cycle as a tiebreaker for prioritization of adult patients to intensive care in an extreme pandemic setting. Details are provided in Additional file 4.

We also present a general description of each of the tiebreakers presented to the public during the deliberation. Details are provided in Additional file 5.

We illustrate below the main quotes according to the emerging themes (Free translation from French to English are indicated between quotation marks).

### Theme 1. Priority to the youngest - life cycle a preferred tiebreaker

Most participants voiced that it is important to give priority to people who still have stages to live through. Some of them stated that younger patients are more likely to do well in intensive care unlike elderly people, probably considering their ability to withstand medical interventions, their ability to recover from them, and, above all, that, in an extreme situation, healthcare resources should be well managed to save more lives. Others indicated the importance of preserving new generations, emphasizing their solidarity with them. Thus, some quotes in favor of the life cycle of Quebec and Ontario’s participants were:

*“I am a woman, I am the oldest, when I hear the extreme and when I was told one day of intensive care,… and when I was operated it took me 2 years to recover because I did not move…I didn’t move and I was practically paralyzed but I say to myself that the life cycle makes me understand that at a given moment, even if the person was extraordinary or like me perfect, I wouldn’t get over it,…here we are in the extremes, it’s not so much that we like the young person or don’t like the young person, it’s precisely the life cycle; at some point we hope that there will be more time for him [the patient] to recover, to sail around the world on a boat with his three children.”* (Participant QC4).

*“My preference is for the life cycle…it’s for sure that I’ll give more chance to a younger one than to an older one; I’d rather prioritize my offspring over myself…”* (Participant QC3).

We observed that some participants did not overly differentiate between two tiebreakers: the life cycle and the absolute age criteria. Participants agreed to prioritize younger patients both indirectly (through life cycle) and directly (through absolute age). This was observed especially in the Ontario group. Their quotes were:

*I am part of the older generation… Say my grandchild or child were up against each other. We’re in the same boat. There would be no question in my own mind, personally and ethically, that they go first before me. No question in my mind. There’s a lot of history of this. If you go back in human history with all these wars and battles, women and children were respected…. I feel very strongly ethically that life cycle and age have to be at the top of this if it comes to the horrible decision that has to be made (who gets treatment and who doesn’t in the ICU). When I look back on that, it sort of re-enforces my feelings on this.* (Participant ON21)

*Absolute age and life cycle should be at the top…* (Participant ON19).

However, we also noted that most participants did realize the difference between these two tiebreakers. One of them remarked that during a life cycle, each stage is different, because each one has its own opportunities. Other participants argued that by prioritizing according to the life cycle, younger people would have the opportunity to go through other stages of their own life cycle and that older people have already had this opportunity.

*I would score life cycle just a little bit higher than absolute age because there are generally things that we do at different stages of our lives. They are similar in age but nobody is exactly the same.* (Participant ON17)

*It’s similar to age but we’re looking at it in bigger chunks and how far they are within life. So, the idea of allowing younger people or people in their earlier stages to experience life.* (Participant ON1)

However, there was also one participant who expressed their opposition to the life cycle considering it subjective, and another participant who argued against both the life cycle and the absolute age criteria, considering both tiebreakers as unfair.

*I think that life cycle is a value judgment as opposed to something that is an objective criterion.* (Participant ON10)

*I disagree with using life cycle for the same reason as age. Because it is not a fair decision.* (Participant ON7)

In relation to the absolute age tiebreaker, for most of the Quebec participants, this was one of the criteria that was considered not very acceptable, since only one participant expressed their acceptance of it. While in Ontario there were more proponents of the absolute age tiebreaker. One of them remarked that taking absolute age into account is important because younger patients would take less time to heal if hospitalized in intensive care, as opposed to older patients:

*“It’s hard, but to 2 cases that are completely equal… you have to choose I think… I think the age of the young person may take precedence”.* (Participant QC14)

*…I think age should be at the top. The biggest reason would be that the older you are, the longer it takes. And the odds are basically against you to begin with. It may not seem fair to you being in the hospital, but those are just facts. You’re older and it definitely takes you longer to heal.* (Participant ON1)

Arguments against the absolute age tiebreaker were also mainly expressed by most participants from Quebec, while in Ontario only a few were against it. The participants who were against absolute age as a tiebreaker argued that it was not an objective criterion, especially if it was considered as the only criterion. Faced with two patients with little difference in age and with the same life prognosis, participants believed that it would be very difficult to make such a tiebreaking decision and that the decision could be biased. Some quotes were:

*“…For me, age is contrary to my values; it’s subjective…”* (Participant QC15).

*“…but a person who is, for example, 39 years old compared to 40 years old as we were talking about earlier, there is really a very thin line between the 2, there is not really a big difference there, it’s really because of that that the absolute age I had a little problem …”* (Participant QC16).

### Theme 2. Randomization - A tiebreaker of last resort

Participants from both Quebec and Ontario voiced their acceptability for randomization as a tiebreaker to be used as a last resort or after considering other tiebreakers. Some participants were against using randomization as a tiebreaker giving the idea that there were other criteria more ethically justifiable than this one.

*“Yes, but with reservations insofar as I understand that we are in a situation where we have exhausted all means; we have untied the knots, I understand, but I still need to have an ethical element for a criterion other than chance alone…”* (Participant QC1).

*…Lottery is the very last, the one you don’t want to use unless you don’t have a choice to consider it.* (Participant ON23)

We also observed that some participants experienced a change of perspective regarding the randomization tiebreaker. They indicated that, at the beginning of the process, it was inconceivable to them to consider a lottery to allocate the only available critical care bed among patients with similar prognosis, but that their perspectives had changed.

*“I was completely against it last week, but as I thought about it…in my opinion, I am divided, but I think that the lottery would be the fairest because there is no longer a social factor, everyone has the same chance.”* (Participant QC13).

*“I was also quite against it, but finally it has really changed. I find that when you look at all the criteria, it is really the one that does not discriminate. Even if I think it’s a bit weird to let chance decide who will live and who will die in certain situations, I still think it’s the fairest…”* (Participant QC11).

On the other hand, some participants from Quebec and Ontario voiced their non-acceptance for this tiebreaker. They argued that considering the lottery as a tiebreaker was an insensitive and risky way to choose between patients because human lives were at stake, and that, when having brilliant minds to reflect on these decisions, the least expected criterion was this one. Others mentioned that chaos would result from making these decisions through randomization alone.

*“I find it totally unacceptable. I think that as we speak there are so many brilliant human beings, there is surely another way to decide because life is not a game; it is important, and I think that using the word lottery kills all the humanity that we can have in each of us.”* (Participant QC16).

*You’d have the chaos. You’d have the cold sense – the decision isn’t made by anybody. It’s like saying that you don’t want to make the decision so you’re just going to roll the dice. It’s too hurtful. I think practically it makes sense but ethically it would be too hurtful.* (Participant ON12)

### Theme 3. The multiplier effect of the most exposed healthcare workers – A tiebreaker of moderate acceptability

In relation to the most exposed healthcare workers tiebreaker, Quebec and Ontario participants expressed a moderate level of acceptability. There were some objections that were stated by participants, such as the uncertainty of returning to work. Although some indicated than even if it is not certain that they will return to work, they would support them anyway. They were more interested in the instrumental value of these workers in the hope that by prioritizing them, they would later be available to save more lives. However, when considering giving priority by reciprocity towards them (only out of gratitude), both Quebec and Ontario participants were not very supportive of the idea.

*…We can’t guarantee that they can save lives later, but we’re already short on nurses and doctors. And so, I would rather take my chances on saving someone who could save lives and is already qualified to save lives even though they don’t end up saving lives than to just eliminate those people. We are already short so why do we want to be even shorter?…* (Participant ON8).

*“Just because they helped us doesn’t mean they can get a higher grade, just because they came out and chose to do the job doesn’t mean we’ll thank them.”* (Participant QC2).

In addition, one participant considered the multiplier effect unacceptable, indicating that it could be considered as a social value of the individual. Conversely, another participant pointed out a difference between the two values, especially during a pandemic, because of the care healthcare personnel provide.

*“I find that healthcare personnel, their multiplier effect a little bit within the social value, like playing with words, I find this unacceptable.”* (Participant QC18).

*“Honestly, healthcare staff is something I would choose; that I distinguish from social value; I really distinguish the level of care that these people provide to the population.”* (Participant QC8).

### Theme 4. Social value of individual - A less acceptable tiebreaker

The social value of an individual was the least accepted tiebreaker among participants from both Quebec and Ontario. Participants considered that the societal value of patients could lead to discrimination due to its inherent subjectivity. We illustrated some of their arguments in the following quotes:

*“We had seen earlier that social value was the one we were least comfortable with, it’s the criterion we can eliminate, it wouldn’t be a choice.”* (Participant QC3).

*…the lowest score possible. I’ll push it even further. Not only is it too subjective but it opens the gate to discrimination…* (Participant ON8).

Some participants considered taking pregnant women into account under the social value of the individual as there would be two lives to save and because it would also include the category of caregivers (those who take care of children or vulnerable people).

*That would have to go under social value. Because pregnancy can be a very wide age group. So, you can’t really do it that way. For social value, you really need to consider the fact that there are 2 lives rather than 1. And one of those lives hasn’t even begun yet. So, they have everything to experience. And you also have a caregiver value in there…* (Participant ON26).

*I think yes, caregivers should be up there at a high number because they have other people depending on them. And if you’re not there, who will take care of them?* (Participant ON13)

## Discussion

This DD enabled us to explore the perspectives of a sample of Quebec and Ontario’s citizens regarding the acceptability of the tiebreakers contained in the COVID-19 pandemic prioritization protocols of their respective provinces. It is noteworthy that, during the training session, participants’ perceptions regarding the most and least acceptable tiebreakers were already apparent. We also noted the difficulty of the participants to accept and discuss this type of topics. Especially because these issues are sensitive, complex and can arouse multiple emotions. We observed that, instead of entering a formal discussion on these criteria, some of them tried to escape the subject. This occurred initially during the deliberation, and it took some time for the deliberators to fully reflect on the real dilemmas of prioritization. We were aware that prioritization in a pandemic crisis context is a difficult subject to deal with. We believe that during this deliberative exercise, participants were also able to understand how complicated it is for the prioritization teams or the clinicians in charge in a stressful pandemic environment to make such decisions.

### Prioritizing the youngest under a combined perspective of equity and equality of chance

One of the tiebreakers that stood out most in terms of acceptance was the life cycle tiebreaker. Quebec participants seemed to have a greater understanding of the ethical values underlying this tiebreaker. However, a few participants from Ontario indicated that they did not perceive any major difference between life cycle and absolute age. This would be in line with other public consultations in which it was not clear under what values the public would prioritize younger patients in the face of equal survival [23, 24]. One of the explanations for this could be the complexity of these ethical issues, which are new to participants and for which it is difficult to detect the nuances.

In the case of the life cycle, participants supported the idea of giving priority to younger patients considering that they have not lived through all the stages of human life, recognizing that each stage of life has its unique characteristics or opportunities. There was also the importance of preserving generations, comparing past times in which priority was given to children and women in catastrophic events or to cope with war, probably also considering their vulnerability. We could also consider it a gesture of solidarity for these vulnerable groups [25]. Thus, participants considered the value of intergenerational equity as relevant in an extreme pandemic context.

Considering the life cycle as a tiebreaker makes equity prevails and favors equality of opportunity for patients to be prioritized. This equality of opportunity with respect to life cycle does not lead to an injustice towards the elderly since they have already had this opportunity at the appropriate time. For some experts, this life cycle criterion is considered an ethically justifiable criterion under extreme conditions [26–28]. Its indirect relationship with chronological age is associated with the passage of time such that when we advance in age, we approach the end of this cycle. For experts, respecting intergenerational equity by considering the life cycle is a human right to respect [29, 30]. One expert suggests that this criterion should be integrated as a first-order criterion in the prioritization and not only as a tiebreaker, which supports its relevance [29]. He also points out that some prioritization protocols were modified based on this criterion to avoid criticism from an ageist perspective.

It was interesting to explore participants’ reflections in relation to these tiebreakers. Their arguments were in line with those of scholars who also suggest that age-related criteria are important to take into consideration when prioritizing in extreme contexts [26–30].

### The egalitarian perspective integrated to the utilitarian perspective

In the search for an egalitarian perspective, participants were unconvinced that the randomization criterion should be a first-order tiebreaker option. Most participants from both provinces disagreed with the randomization criterion as they did not consider it reasonable to use when making life or death decisions. We understand that the participants were referring to the consequences that would result from taking such an important decision ‘lightly’ e.g., tossing a coin, without considering other factors important to them (such as age-related aspects for example). For them, the lottery was an inappropriate way to make this decision, as if people were material goods. From the beginning of the deliberation, the participants diverged regarding the acceptability of this criterion. However, at the end of the deliberative process, many participants indicated that it could be an option to be implemented, but after considering other tiebreakers such as life cycle. This would be a way to integrate both equality and equity in a utilitarian perspective. It is coherent with previous ethical suggestions [31].

### Their instrumental value more than reciprocity for the most exposed healthcare workers

When considering healthcare workers as the most exposed group during this pandemic, some participants voiced giving them priority as a tiebreaking criterion. This is due to the possibility that healthcare workers could continue to save more lives after their recovery, especially considering the scarcity of personnel in healthcare institutions and public services. In other words, prioritizing healthcare workers could lead to a better outcome for the population. This is also consistent with results from other public consultations conducted during this pandemic [32–34]. The reciprocity towards healthcare workers was poorly supported in the sense of giving them priority for having exposed their lives for the benefit of others, in gratitude for their sacrifices. They considered that not only this group of workers were owed thanks for their labors during this pandemic. Likewise, others indicated that healthcare workers are already aware of the risks associated with their work. In a public deliberation conducted in North America including caregivers and patients, the latter supported reciprocity towards the most exposed healthcare professionals [13]. This shows that participants’ values can be influenced by their healthcare experiences and sociocultural background.

From a utilitarian perspective, in times of health crisis, the consideration of prioritizing healthcare professionals over other groups of workers as they are the most exposed would be based mainly on the need to alleviate the ravages of this type of situation. If it were another type of catastrophe or critical situation for society, the prioritization of other groups of essential workers would probably be contemplated. In the literature, we can find other examples of the relevance of the social contract between healthcare workers and the population [35]. It should be noted that healthcare professionals have a duty of care and a duty to treat the population, but that these obligations also have their limits. These limits would be associated with the prevention of abuse and mistreatment at work, as the work of these healthcare workers is risky and exhausting, the boundary between risky work and abuse is easy to cross [36]. Mortality in this group of workers was also high during the first and hardest pandemic waves, most due to exposure to the virus. Work absenteeism was also high due to healthcare workers suffering from the disease on more than one occasion due to the different viral variants, among other reasons [37]. As the shortage of these workers increased, the overburden on labor was carried by those still on the viral battlefield.

To ensure that the population respected the health measures put in place by governments, most of the public and private workers were working remotely. However, healthcare workers, the most exposed group, were working on-site with schedules and shifts beyond those initially established in their employment contract. The physical and mental workload was disproportionate, a situation that would be considered unfair but unavoidable in the face of a pandemic event.  It could have been considered that society has a duty of care towards them as well. However, it seems that this duty has not been fully understood or perhaps will continue to be ignored.

### The controversial social value of the individual

It is noteworthy that, from the beginning of the training until the day of the deliberation, most of the participants did not agree with considering the value of the individuals based on their socio-economic status or functions in society. For participants, it was not fair to consider these non-clinical patient characteristics because not everyone has had the same opportunities and preferences in life. Making an exception for pregnant women was supported by some participants, considering this argument both a social and instrumental value. One possible interpretation is that participants had in mind the healthy pregnant woman who could give birth, not the one with COVID-19 whose prognosis is bleak despite carrying life inside her. They were possibly referring to the symbolic aspect of a pregnant women. In analytical psychology, archetypes refer to an inward image at work in the human psyche [38]. When discussing a hypothetical situation, it can be demanding, if not impossible, to measure its real impact. The interference of a powerful, untouchable symbol such as the pregnant woman may have influenced the meaning of the discussions among the participants.

We could say that the value of familism was considered by only a few participants. Under the familism perspective, parents or caregivers of children or dependents would be prioritized, including pregnant women. Our findings contrast with a study conducted during the influenza pandemic where most participants gave high importance to familism [39].

The social value of the individual had also been rejected in other public deliberations, for example in Thailand [12]. We believe that this value will continue to be a matter of discussion for experts and non-experts, although a tendency for its low acceptance has been observed [39–41].

### Limitations

Our study has several limitations that deserve mention. First, the present study was solely conducted online and not in person, due to the health measures in place in both provinces at the time of the training and deliberation process. This modality proved to be a success for both the team and the participants. Despite some technical limitations, we consider that an online DD is a promising alternative in cases where it cannot be carried out in person. Few studies have been conducted using this modality. A number of technical aspects and other adjustments must be considered to carry them appropriately. Second, our sample was not representative of the two communities studied, which limits our ability to generalize the present results. Third, we were only able to qualitatively analyze the records of the deliberation session and the observation notes, as the records of the training day were incomplete. Fourth, some participants contacted by the INM were not able to connect to the Zoom system because of internet failures due to bad weather conditions on the day of the training session, and another for emergency reason. This ultimately reduced the number of study participants. Finally, in this DD, to reach a consensus was optional. Consensus is not mandatory in a DD since experts have noted the difficulty of obtaining it due to the heterogeneity of perspectives and values of the communities.

Despite these limitations, we believe that the strength of this study is that it is one of the first citizen deliberations undertaken to better understand the perspectives and acceptance of tiebreakers of the COVID-19 prioritization protocols. The information gathered from participants may be useful for optimizing the protocols in extreme pandemic contexts.

## Conclusions

This paper summarizes the perspectives of a sample of Quebec and Ontario citizens who participated in an online DD regarding the prioritization protocols for accessing intensive care, specifically on tiebreaker criteria to consider in the context of an extreme pandemic. Participants reflected on how best to allocate scarce healthcare resources in an extreme situation in the event of encountering patients with similar survival prognosis. A utilitarian perspective prevailed, but with the integration of the egalitarian and equity perspective. The tiebreaker with the highest acceptability in both groups was the life cycle criterion, emphasizing respect for intergenerational equity. Most of the public consultation conducted during this COVID-19 pandemic converge with our findings. Strong support for the prioritization of younger patients suggests that age, both indirectly and directly, is relevant to citizens for allocating scarce healthcare resources. The tiebreakers associated with the profession of individuals or their usefulness in society were not well accepted by participants. Although no final consensus was reached at the end of the deliberation, there was a tendency towards a moderate acceptance for the instrumental value of the most exposed healthcare workers and for randomization, but a very low level of acceptance for considering the social value of the individual. The public needs more information about prioritization in the context of pandemic crisis, their perspectives on tiebreakers should be further explored.

### Electronic supplementary material

Below is the link to the electronic supplementary material.

Supplementary Material 1

Supplementary Material 2

Supplementary Material 3

Supplementary Material 4

Supplementary Material 5

## Data Availability

The datasets generated and/or analysed during the current study are available from the corresponding author upon reasonable request.

## Abbreviations

COVID-19: Coronavirus disease 2019
INM: Institut du Nouveau Monde
