# Supplementary material for: What are the views of Quebec and Ontario citizens on the tiebreaker criteria for prioritizing access to adult critical care in the extreme context of a COVID-19 pandemic?
Source: BMC Med Ethics. 2024 Mar 19;25:31. doi: 10.1186/s12910-024-01030-2 (PMC10949716; doi:10.1186/s12910-024-01030-2)
Supplement: Supplementary file 1 — Supplementary Material 1 [file 12910_2024_1030_MOESM1_ESM.docx]

**Additional file 1.** Democratic Deliberation Program for participants

| **DAY 2. DELIBERATION SESSION - JUNE 4, 2022 (8:30 - 12:00)**  8:30 Welcome  **8:40** Discussion - Feedback on the first session  **9:10** Small group discussion  ● Reflect on the benefits and concerns regarding the protocols  ● Reflection on the prioritization secondary criteria (Tiebreakers)  ● Discussion on the relevance  ● Question on acceptability of tiebreakers  ● Wrap-up:  **How do we choose who would have access to intensive care units in an extreme pandemic setting? Under what criteria?**  ● A facilitated discussion around two questions on other ethical issues  **10:45** Break and return to large group  **11:00** Sharing and discussion  **12:00** End  Source: INM -Adaptation of the original program |
| --- |
