# Supplementary material for: What are the views of Quebec and Ontario citizens on the tiebreaker criteria for prioritizing access to adult critical care in the extreme context of a COVID-19 pandemic?
Source: BMC Med Ethics. 2024 Mar 19;25:31. doi: 10.1186/s12910-024-01030-2 (PMC10949716; doi:10.1186/s12910-024-01030-2)
Supplement: Supplementary file 2 — Supplementary Material 2 [file 12910_2024_1030_MOESM2_ESM.docx]

**Additional file 2.** Fact sheet on main terms used in prioritization for participants

| **Triage or prioritization** These are ways of allocating healthcare and services when they are scarce. Examples include first-come, first-served, lottery (random), or use of a prioritization protocol, among others.  **Extreme Pandemic Context**  An extreme situation in which many people are affected by a serious contagious disease (e.g., caused by a virus), but health services are unable to treat all of them because resources (human or material) are limited. As a result, many of these people may die due to lack of care.  **Intensive care (IC)** This is specialized care for patients with the most serious conditions, such as those who require equipment like artificial ventilators or specialized monitoring.  **Prioritization protocols for access to adult critical care in the extreme pandemic setting**  These are guides to determine which patients would benefit most from this care when it is not available, in an attempt to save as many lives as possible.  **Lottery or chance**  Establishes prioritization in access to care based on a random process like a lottery.  **First come, first served**  Prioritizes patients based on their order of arrival at the hospital.  **Principles and values** These are our beliefs about doing the right thing (what we think is right) for ourselves and our community. We all have different values and principles that govern how we are and how we behave. For example: benefit maximization, equality, fairness, reciprocity, responsibility, etc.  **Benefit maximization** This principle aims at a common good. For example: to do the greatest good for the greatest number of people.  **Equality**  This principle aims at equal opportunity for all patients regardless of their specific characteristics or needs. Chance (or the lottery) is one way to apply this principle.  **Equity**  This principle aims to give priority to the most needy and vulnerable patients, taking into account their clinical and non-clinical characteristics so as not to disadvantage them. The life cycle is an application of this principle.  **Tiebreaker** These are criteria used when, for example, two patients have similar chances of survival. The criteria are used to break the tie between two patients who would be on the same level. For example, life cycle, instrumental value, caregiver, lottery or social value are tiebreakers.  **Life cycle** It prioritizes the youngest patient because he or she has not had the chance to live through several life stages.  **Absolute age**  It prioritizes the patient based on age alone, favoring the youngest.  **The multiplier effect**  It prioritizes essential personnel, because by recovering they can continue to save more lives. This category includes firefighters, paramedics, police, nurses, doctors, etc.  **Healthcare personnel** It prioritizes the most exposed health personnel during the pandemic, as a reciprocity because the task is risky and to thank them for their effort.  **Social value** It prioritizes the patient who can be more useful to society after his recovery, for example: a mother/father, a scientist, a politician, etc.  **Clinical Survival Criteria**  These are criteria that physicians use to determine the likelihood of survival (prognosis) of a critically ill patient. They use their clinical judgment and evidence-based scores/scales. Prioritization protocols contain these criteria.  Source: Adaptation of general research project |
| --- |
