# Supplementary figures and images for: What are the views of Quebec and Ontario citizens on the tiebreaker criteria for prioritizing access to adult critical care in the extreme context of a COVID-19 pandemic?
Source: BMC Med Ethics. 2024 Mar 19;25:31. doi: 10.1186/s12910-024-01030-2 (PMC10949716; doi:10.1186/s12910-024-01030-2)

**Additional file 3.** Intercoder reliability


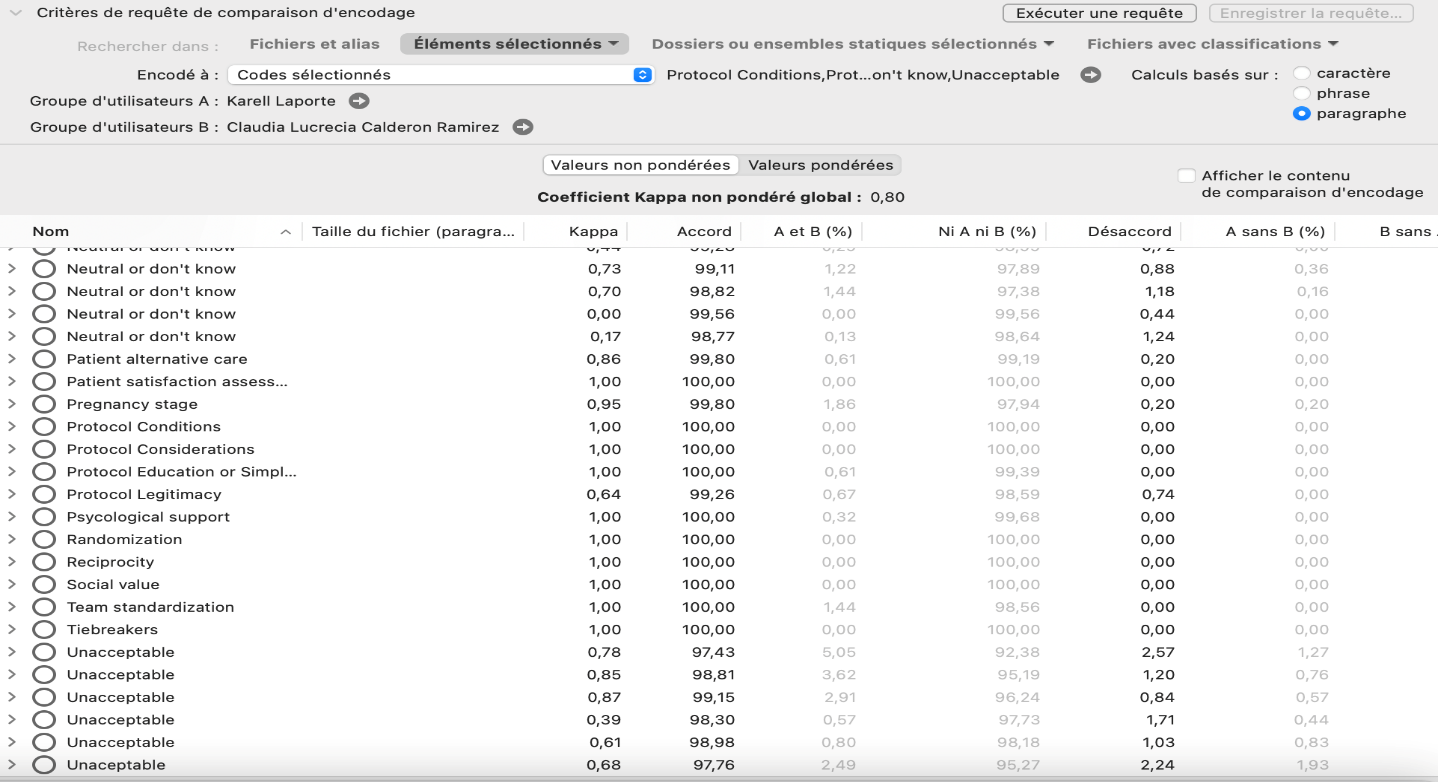

Supplement: Supplementary file 3 — Supplementary Material 3 [file 12910_2024_1030_MOESM3_ESM.docx]
