# Supplementary material for: What are the views of Quebec and Ontario citizens on the tiebreaker criteria for prioritizing access to adult critical care in the extreme context of a COVID-19 pandemic?
Source: BMC Med Ethics. 2024 Mar 19;25:31. doi: 10.1186/s12910-024-01030-2 (PMC10949716; doi:10.1186/s12910-024-01030-2)
Supplement: Supplementary file 4 — Supplementary Material 4 [file 12910_2024_1030_MOESM4_ESM.docx]

**Additional file 4.** Diagram of coding comparison on the Life cycle acceptability according to participants


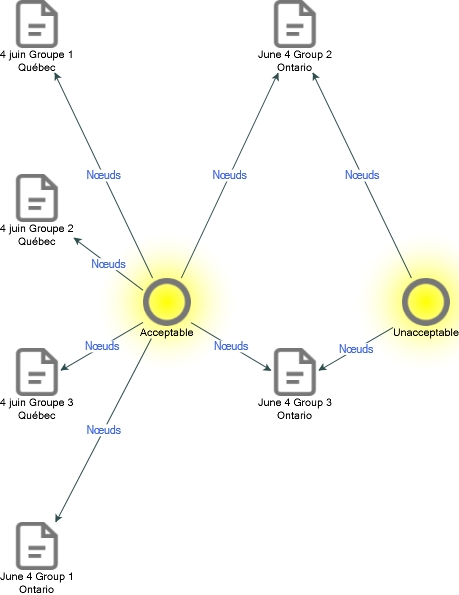


Source: *NVivo 2022* software
