# Supplementary material for: What are the views of Quebec and Ontario citizens on the tiebreaker criteria for prioritizing access to adult critical care in the extreme context of a COVID-19 pandemic?
Source: BMC Med Ethics. 2024 Mar 19;25:31. doi: 10.1186/s12910-024-01030-2 (PMC10949716; doi:10.1186/s12910-024-01030-2)
Supplement: Supplementary file 5 — Supplementary Material 5 [file 12910_2024_1030_MOESM5_ESM.docx]

**Additional File 5**

**Table. Tiebreakers the most frequently found in prioritization protocols COVID-19**

| **Tiebreakers** | **Concept** | **Characteristics** |
| --- | --- | --- |
| **Absolute age** | Prioritize patients only by their age favoring the youngest. | A controversial criterion. Acceptable if not isolated - other patient conditions should be considered. |
| **Life cycle** | It gives priority to children, adolescents and young adults who have not yet had the opportunity to experience the other stages of their lives. | It is justified by intergenerational equity. |
| **Multiplier effect** | It gives priority to the most exposed healthcare personnel, to preserve its services for the benefit of the population. | It is justified by solidarity, and “narrow social utility”. |
| **Reciprocity** | Prioritizes the most exposed healthcare workers out of gratitude for their altruistic and risky work during the pandemic. | It is justified by gratitude and solidarity. |
| **Randomization** | It grants priority based on luck obtained randomly (by lottery or a coin toss) | It is representative of equality. Easy and quick to apply. |
| **Social value** | It gives priority to people who, due to their family status or their role in society, are difficult to replace. | A controversial criterion.  It is justified by reciprocity and solidarity. |
| **First come, first served principle** | It grants priority according to the order of arrival of patients in a hospital or health institution. | In a healthcare crisis, it can benefit the most socioeconomically advantaged. Little used as a tiebreaker. But in a conventional situation represents equality. |

Source: Adaptation of general research project.
